# Impact of the COVID-19 pandemic on maxillofacial trauma surgery in Germany - implications from the national DRG database

**DOI:** 10.1007/s10006-024-01248-9

**Published:** 2024-04-11

**Authors:** Axel Meisgeier, Simon Pienkohs, Laura Moosdorf, Andreas Neff

**Affiliations:** 1 Department of Oral and Craniomaxillofacial Surgery, Faculty of Medicine, UKGM GmbH, University Hospital Marburg, Philipps University, 35043 Marburg, Germany; 2 Center for Orthopaedics and Trauma Surgery, Faculty of Medicine, UKGM GmbH, University Hospital Marburg, Philipps University, Marburg, Germany

**Keywords:** COVID-19, Fracture epidemiology, Maxillofacial fracture, SARS-CoV-2, Traumatology

## Abstract

**Purpose:**

The COVID-19 pandemic has affected the personal and social lives of millions of people and also impacted the etiological factors of midfacial trauma such as falls, interpersonal violence or traffic accidents. The aim of this study was to analyze the influence of the COVID-19 pandemic on maxillofacial trauma surgery in the German healthcare system.

**Methods:**

Nationwide data regarding the national diagnosis-related-group (DRG) inpatient billing system used in all German hospitals was received from the German Federal Statistical Office. Various trauma-associated procedures of the Operation and Procedure Classification System (OPS), a German modification of the International Classification of Medical Procedures (ICPM), were statistically associated with different epidemiological factors between 2012 and 2021.

**Results:**

A statistically significant decrease (*p* < 0.05) in surgeries regarding maxillofacial fractures was registered during the years 2020 and 2021. Young male patients had the largest decline in maxillofacial trauma surgeries during this period (*p* < 0.05). In contrast. elderly patients 80 years and older showed a dramatic increase in the frequency of fractures in both the midface and the mandible (*p* < 0.05).

**Conclusions:**

During the COVID 19 pandemic there has been a shift in the number, composition and etiology of maxillofacial fracture surgeries. Measures of social distancing and personal risk avoidance had a societal positive effect on the frequency of facial injuries. This stands in contrast to the drastic increase in fractures of elderly people who should be protected primarily by the measures taken. These results can help to understand these influences better in future pandemics.

**Trial registration:**

German Clinical Trials Register No: DRKS00032778.

## Introduction

The COVID-19 pandemic caused by the Severe Acute Respiratory Syndrome Coronavirus 2 (SARS-CoV-2) has had an enormous impact on the life, behavior and habits of each individual as well as on the coexistence of society in general. Both personal and governmental measures have been taken worldwide to curb the spread of the virus and, in particular, to protect risk groups [1]. Measures of social distancing included, among other things, the ordering of home quarantines, residence regulations, nationwide curfews, cancellation of community events and the reduction of social contacts. Elective health treatments have been temporarily suspended or postponed in favor of caring for affected patients and conserving human resources [2]. The impact of all the measures taken on the incidence and epidemiology of non-elective care in the field of facial traumatology, has not yet been fully clarified and would prove the success of the measures taken regarding the preservation of medical resources.

Maxillofacial fractures are among the most common bone fractures and patients with maxillofacial fractures constitute a large percentage of all patients in maxillofacial departments in Germany [3]. Facial bone fractures involve the zygomatic complex, the orbital walls, the maxilla and mandible, the teeth and the paranasal and frontal sinuses. Traumatic facial injuries are particularly common among young adult males [4, 5]. The most common causes are traffic accidents, falls, physical violence and sports injuries. These are subject to strong seasonal trends and fluctuations [3, 6, 7]. Open reduction and internal fixation has become the standard procedure for fractures of most facial regions. Since the evidence for the superiority of surgical treatment in the area of mandibular condylar process fractures has been proven in the last two decades, conservative treatment with closed reduction and maxillomandibular fixation still plays a certain role [8–10].

Data from a single center study in Germany suggest a decline in frequency of traumatological cases between 2019 and 2020 in both facial fractures and facial soft tissue injuries [11]. Local single center studies and regional multi center studies from different countries and continents have also shown a change in volume and a shift in the type and cause of facial injuries, despite to our knowledge there is no nationwide investigation representing a central European country like Germany [12].

The purpose of this study was to examine the nationwide impact of the COVID-19 pandemic on patients in need of surgical management of facial fractures in Germany. For this purpose, the regional, gender-specific and age-related distribution of patients with facial fractures who required inpatient open reduction and internal fixation in various anatomical regions of the midface and mandible were evaluated.

## Materials and methods

The national diagnosis-related groups (DRG) inpatient billing system includes data from all hospitals in Germany that use the DRG system. More than 99% of inpatient treatments in Germany are covered. Hospitals are required by law to provide comprehensive information about hospital care, including patient demographics, diagnoses, comorbidities, complications and procedures.

Surgical procedures from the years 2012 to 2021 were coded according to the Operation and Procedure Classification System (OPS), a German modification of the International Classification of Medical Procedures (ICPM). All diagnoses were coded according to the German version of the International Classification of Diseases and Related Health Problems, 10th Edition (GM ICD-10). Detailed lists of all procedures regarding facial fractures per year (coded 5-760, 5-761, 5-762, 5-763, 5-764, 5-765, 5-766 and 5-767 in the German procedure classification OPS) were provided by the German Federal Statistical Office (Statistisches Bundesamt - Destatis. Genesis-Online. Data licence by-2-0). The dataset contains all inpatient procedures regarding maxillofacial fractures performed in Germany within the observational period classified by year, gender, age and federal state. Maxillofacial procedures per year (PPY) were calculated and reported as median and range. In addition, population-adjusted rates of maxillofacial trauma surgeries per 100 000 inhabitants were calculated using population data also provided by the German Federal Statistical Office. In order to overcome seasonal influences, the fracture incidence was analyzed per year. Procedures were grouped according to the years 2012–2019 as pre-COVID years and 2020–2021 as COVID years. Cumulative incidences of COVID-19 were provided by the Robert-Koch-Institut [13]. The normality of the distribution of continuous variables was tested by Kolmogorov-Smirnov test. Continuous variables with normal distribution were presented as mean and standard deviation; non-normal variables were reported as median and range. Means of 2 continuous normally distributed variables were compared by independent samples Student’s t-test. Mann-Whitney U test was used, respectively, to compare means of variables with group sizes smaller than five or not normally distributed. Categorical data was presented through frequencies and percentages. The differences were considered significant at *p* < 0.05. Two-sided p-values were presented unless otherwise noted. Statistical analysis was performed using IBM SPSS Statistics Version 29.0 (IBM Deutschland GmbH, Böblingen, Germany). This trial was registered at German Clinical Trials Register (https://www.drks.de) No: DRKS00032778.

## Results

### Epidemiology, age and gender

**Fig. 1:**
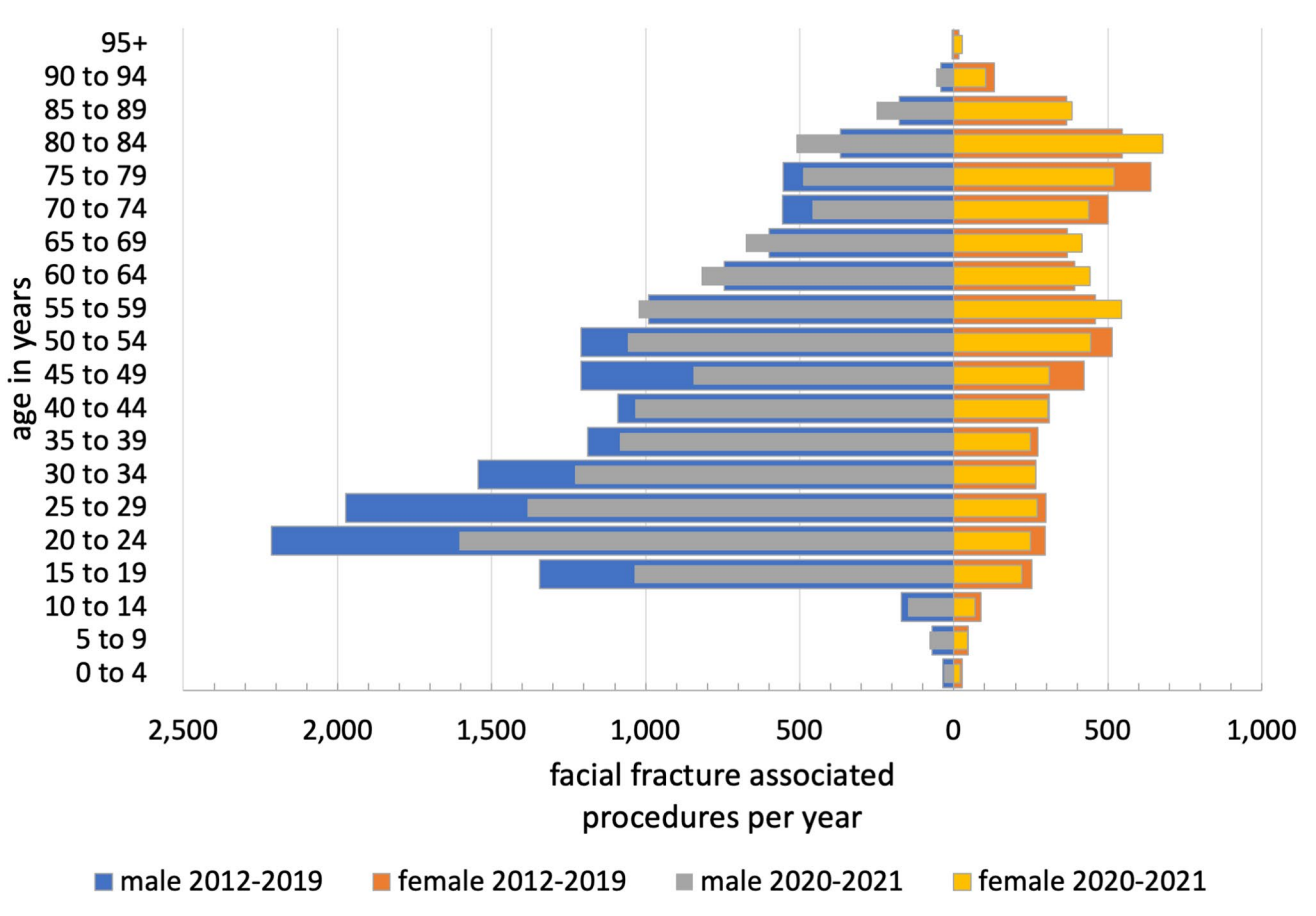
Age-gender-diagram of facial fracture associated procedures per year before and after the COVID-19 outbreak

In the years 2012–2019 a median of 22,480 (21,672–23,199) maxillofacial trauma procedures per year (PPY) were registered. During the COVID-19 outbreak in 2020 and 2021, there was a statistically significant decrease to 19,834 PPY (19,549–20,118). This means a relative decrease of 10.7% (*p* < 0.05) (Table 1). In males the number of maxillofacial trauma procedures decreased from 15,929 PPY (15,688–17,088) to 13,831 PPY (13,593–14,069), viz. -13.2%, (*p* < 0.05). The number of treatments in females decreased from 6172 PPY (5984–6520) to 6003 PPY (5956–6049), viz. 2.7%, not significant (n.s.), respectively (Fig. 1). Gender distribution shifted significantly from 71.7% in males and 28.3% in females to 69.7% and 30.3%, respectively (*p* < 0.05). The mean age of the patients increased significantly from 45.8 ± 21.6 years before the pandemic to 48.4 ± 22.0 years during the 2020–2021 pandemic years (*p* < 0.05). In children aged 0–14 years treatment frequency decreased by 9.3% from 437 PPY (396–509) to 396 PPY (385–403) (n.s.). Young adolescents and adults aged 15–34 years showed the largest decrease in facial trauma surgery by 22.5% from 8187 PPY (7745–9210) to 6260 PPY (6240–6280, *p* < 0.05). In the age group 35–59 years the number of fracture repairs decreased from 7663 PPY (7542–7954) to 6896 PPY (6698–7095), corresponding to a decrease of 9.9% (*p* < 0.05). In the age group 60–79 years surgical fracture reduction decreased only slightly from 4357 PPY (4115–4700) to 4261 PPY (4175–4346) by 1.8% (n.s.). Remarkably, the incidence of fracture repairs in the age group over 80 years increased against the trend by 23.5% from 1653 PPY (1411–1980) to 2023 PPY (2012–2033) (*p* < 0.05). Overall the median annual number of inpatient facial injury associated diagnoses (ICD10-S01 - ICD10-S02) also including soft tissue injuries and conservative inpatient treatment decreased from 55,401 between 2012 and 2019 to 44,469 in 2020 and 2021 (-19.7%; *p* < 0.05).

**Table 1:** Median numbers and range of facial fracture associated procedures per year prior to and after the COVID-19 outbreak in Germany (p-value according to * Mann-Whitney-test and ° student’s t-test)

	Pre COVID-19 2012 − 2019	COVID-19 2020 − 2021	Percent change	*p*-value
All facial fracture procedures	22,204 (21,672–23,199)	19,834 (19,549–20,118)	-10.7%	< 0.05*
**Gender** Male Female Distribution male / female	15,929 (15,688–17,088) 6172 (5984–6520) 71.7% / 28.3%	13,831 (13,593–14,069) 6003 (5956–6049) 69.7% / 30.3%	-13.2% -2.7%	< 0.05* 0.09* < 0.05*
**Age** Mean ± standard deviation 0–14 years 15–34 years 35–59 years 60–79 years Over 80 years	45.8 ± 21.6 437 (396–509) 8187 (7745–9210) 7663 (7542–7954) 4357 (4115–4700) 1653 (1411–1980)	48.4 ± 22.0 394 (385–403) 6260 (6240–6280) 6896 (6698–7095) 4261 (4175–4346) 2023 (2012–2033)	-9.3 **-22.5%** **-9.9%** -1.8% **+ 23.5%**	**< 0.05°** 0.18* < 0.05* < 0.05* 0.09* < 0.05*
**fracture of the midface**	**8639** **(8412–9208)**	**7560** **( 7412–7699)**	**-14.3%**	< 0.05*
Fracture of the lateral Midface 5–760	5594 (5264–6126)	4648 (4586–4710)	-20.3%	< 0.05*
Fracture of the central Midface 5-761	1409 (1298–1483)	1251 (1228–1273)	-12.6%	< 0.05*
Fracture of the central and Lateral upper midface 5-762	822 (736–961)	822 (815–828)	-0.1%	0.99*
Combined and complex Midfacial fractures 5-763	864 (766–916)	840 (792–888)	-2.9%	0.71*
**Fracture of the mandible**	**6238** **(6121–6654)**	**5878** **(5829–5926)**	**-6.1%**	< 0.05*
Fracture of the mandibular body 5-764	3530 (3301–3850)	3333 (3250–3416)	-5.8%	0.09*
Fracture of the mandibular Ramus and condylar process 5-765	2693 (2521–2826)	2545 (2510–2579)	-5.9%	0.09*
**Fracture of the orbital wall 5-766**	**6725** **(6466–7112)**	**5951** **(5847–6055)**	**-13.0%**	< 0.05*
**Fracture of the frontal sinus 5-767**	**553** **(499–633)**	**445** **(438–452)**	**-24.2%**	< 0.05*

### Fracture localization

**Fig. 2:**
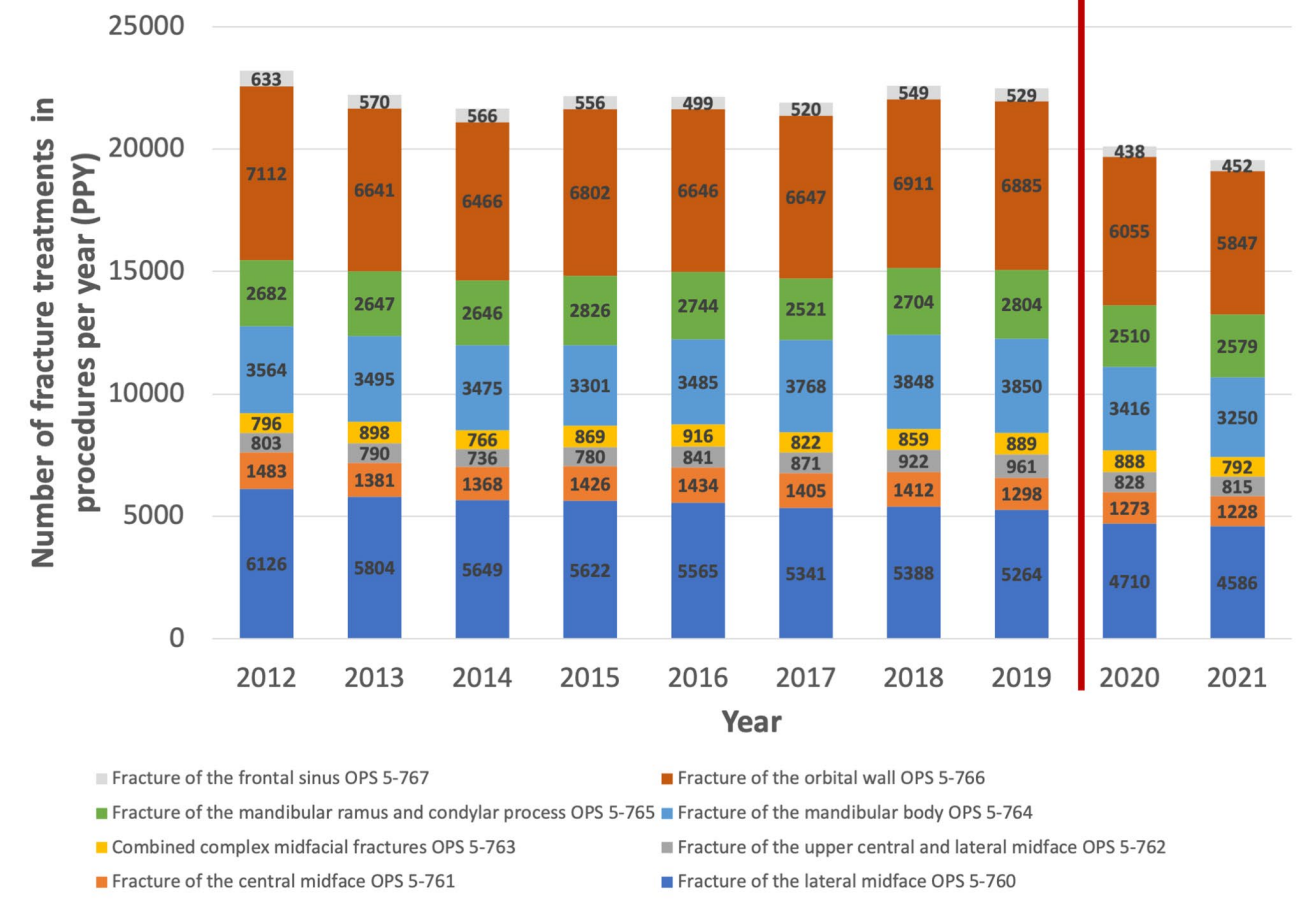
Total numbers of facial fracture associated procedures per year from 2012 to 2021. There were statistically significant changes found after the COVID-19 outbreak (2020–2021) for midfacial fractures, fractures of the mandible, orbital wall fractures and fractures of the frontal sinus (*p* < 0.05, Mann-Whitney-test)

The number of procedures for lateral midfacial fractures (OPS 5-760) decreased from 5594 PPY (5264–6126) to 4648 PPY (4586–4710), a decrease of 20.3% (*p* < 0.05, Fig. 2). The treatment frequency of central midface fractures (OPS 5-761) decreased by 12.6% (*p* < 0.05) from 1409 PPY (1298–1483) to 1251 PPY (1228–1273). The number of mandibular fracture repairs decreased from 6238 PPY (6121–6654) to 5878 PPY (5829–5926) corresponding to a decrease of 6.1% (*p* < 0.05). Reduction of orbital wall fractures decreased by 13% from 6725 PPY (6664–7112) to 5951 PPY (5847–6055) (*p* < 0.05). The surgical treatment of frontal sinus fractures decreased by 24.2% from 553 PPY (449–633) to 445 PPY (438–452) (*p* < 0.05). The frequency of complex and combined midfacial fractures did not show any significant changes during the COVID-19 outbreak. In comparison changes within the pandemic were weak with an overall decrease of 2.8% between 2020 and 2021. Only fractures of the mandibular ramus and condylar head (+ 2.7%) and fractures of the frontal sinus (+ 3.2%) showed a slight increase between 2020 and 2021.

**Fig. 3:**
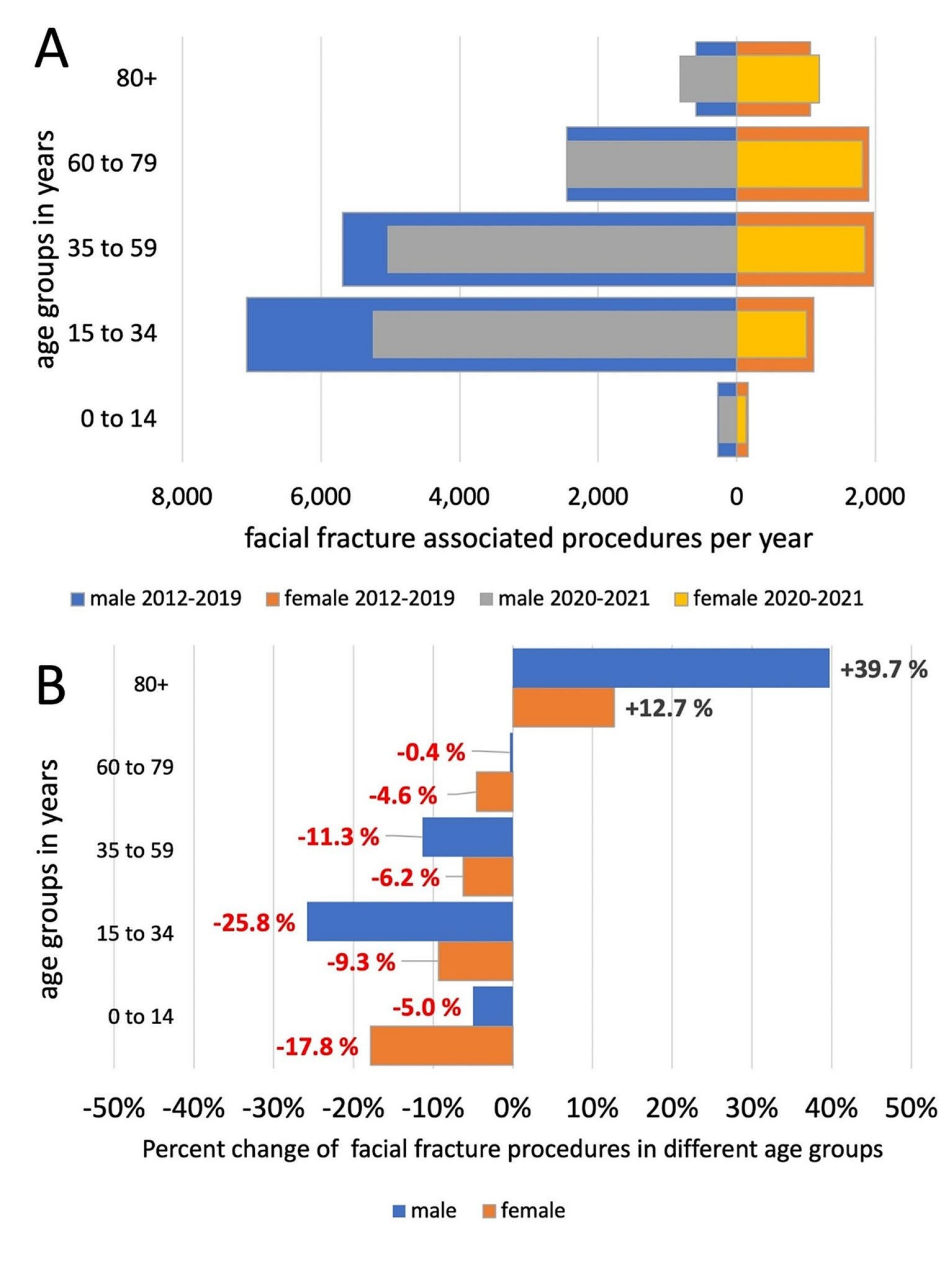
**(A)** Absolute number of facial fracture associated procedures prior to and after the COVID-19 outbreak in the different age groups **(B)** Relative percent change of facial fracture associated procedures prior to and after COVID-19 outbreak in different age groups

In our study, young adolescent and adult males from 15 to 34 years are the main group suffering from facial fractures, representing 32% of all registered maxillofacial trauma procedures. During the COVID-19 outbreak there was the highest absolute and relative decrease from 7076 PPY to 5688 PPY (-25.8%, *p* < 0.05) in this subgroup and representing 58,6% of the overall decline in maxillofacial fracture procedures (Fig. 3). The second most affected group, males aged 35–59, showed the second largest absolute decrease from PPY 5688 to PPY 5044 (-11.3%, *p* < 0.05). Both males and females over 80 years are the only subgroups showing an increase in facial fracture associated procedures from 593 PPY to 828 PPY (+ 39.7%, *p* < 0.05) in males and from 1060 PPY to 1195 PPY in females, respectively (+ 12.7%, *p* < 0.05).

### Regional differences

**Fig. 4:**
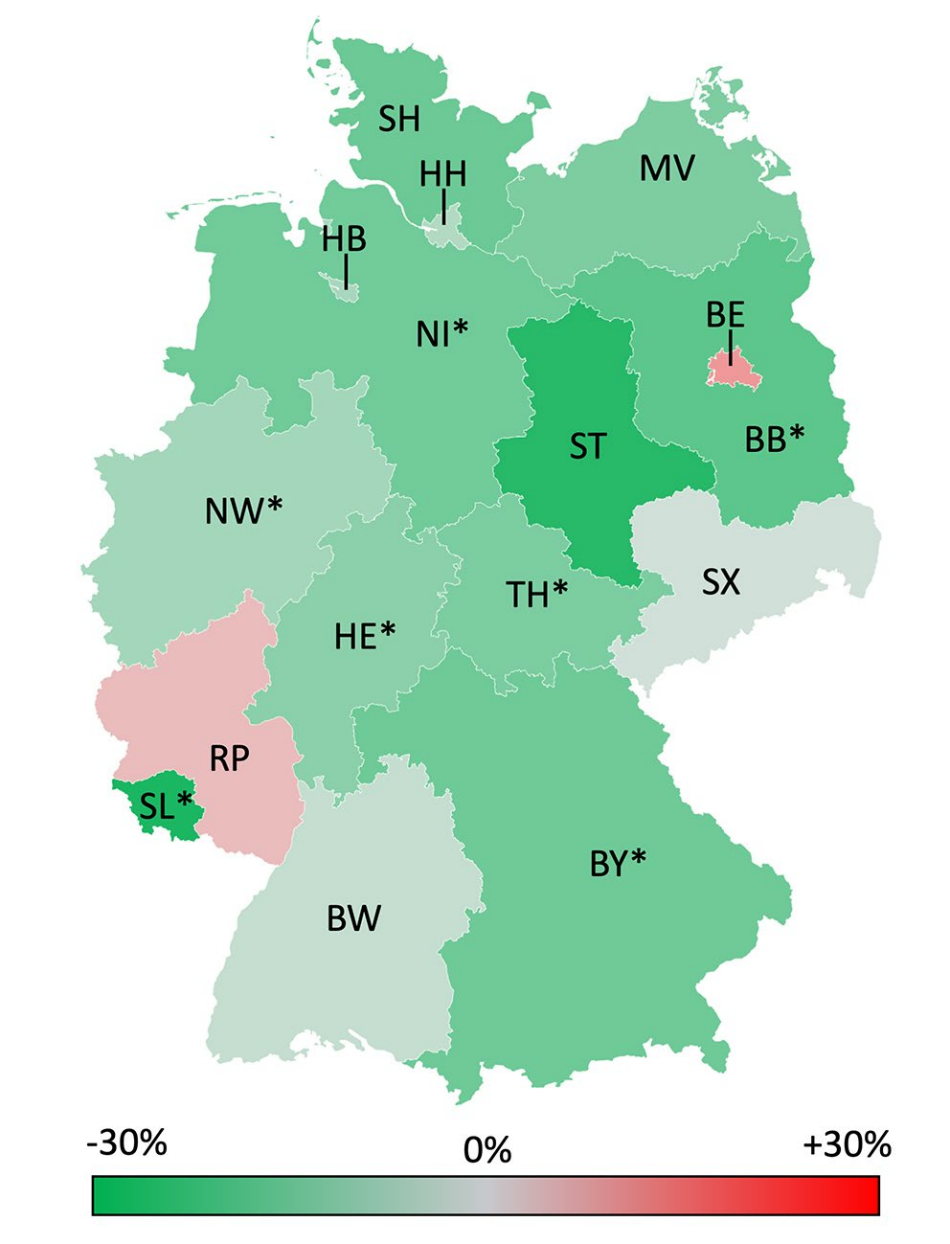
Percentage change of the annual incidence of facial fracture associated procedures after the COVID-19 outbreak (2020–2021) compared to the pre COVID-19 era (2012–2019) in the different federal states. *Significant change (*p* < 0.05, Mann-Whitney-test); SH: Schleswig Holstein; MV: Mecklenburg-Vorpommern; HH: Hamburg; HB: Bremen; NI: Lower saxony; BE: Berlin; BB: Brandenburg; ST: Saxony-Anhalt; NW: North Rhine-Westphalia; HE: Hesse; TH: Thuringia; SX: Saxony; RP: Rhineland-Palatinate; SL: Saarland; BW: Baden-Wurttemberg; BY: Bavaria

Looking at the 16 federal states individually the number of maxillofacial trauma surgery procedures ranges from 19.0 (Brandenburg) to 57.8 (Bremen) per 100 000 inhabitants prior to COVID-19 (Table 2; Fig. 4). During the pandemic there was a decline in facial fracture surgery found in 14 of 16 federal states, 8 of which were statistically significant ranging from − 2.9% in Saxony to -26.3% in Saarland. An increase in interventions can be seen in Rhineland-Palatinate (+ 5.5%) and Berlin (+ 10%), both without statistical significance. There was no correlation to regional cumulative incidence of COVID-19.

**Table 2:** Regional rates of maxillofacial trauma surgery procedures per 100 000 inhabitants in all 16 federal states of Germany prior to and after COVID-19 outbreak (* *p*-value according to Mann-Whitney-test)

	Pre COVID-19 2012 − 2019	COVID-19 2020 − 2021	percent change	*p*-value
Germany	26.3 (25.7–27.5)	23.5 (23.2–23.8)	-10.7%	0.04*
Baden-Württemberg	22.7 (22.2–24.8)	21.7 (20.7–22.7)	-4.4%	0.15*
Bavaria	24.2 (22.4–26.9)	20.4 (19.8–20.9)	**-15.7%**	0.04*
Berlin	28.4 (27.6–33.8)	31.2 (30.4–20.0)	+ 10.0%	0.40*
Brandenburg	19.0 (18.0–24.3)	15.6 (15.6–15.7)	**-17.8%**	0.04*
Bremen	57.8 (49.1–76.2)	53.1 (53.1–53.1)	-8.1%	0.36*
Hamburg	54.3 (49.8–62.5)	50.7 (50.1–51.3)	-6.8%	0.18*
Hesse	25.7 (23.6–29.1)	22.6 (22.0–23.2)	**-12.0%**	0.04*
Mecklenburg-Vorpommern	29.9 (24.5–38.8)	25.8 (24.1–27.5)	-13.7%	0.19*
Lower Saxony	24.1 (21.6–27.0)	20.4 (19.8–21.1)	**-15.2%**	0.04*
North Rhine-Westphalia	26.7 (26.2–28.7)	24.4 (23.9–24.9)	**-8.6%**	0.04*
Rhineland-Palatinate	21.0 (19.7–23.3)	22.2 (21.2–23.2)	+ 5.5%	0.5*
Saarland	34.8 (29.7–40.0)	25.7 (25.7–25.7)	**-26.3%**	0.04*
Saxony	31.8 (28.9–35.9)	30.8 (28.9–32.8)	-2.9%	0.53*
Saxony-Anhalt	28.7 (22.2–32.5)	21.5 (20.6–22.5)	-24.9%	0.09*
Schleswig-Holstein	21.4 (18.4–25.0)	18.0 (17.2–18.8)	-16.0%	0.09*
Thuringia	40.1 (36.2–44.5)	34.5 (33.7–35.4)	**-13.8%**	0.04*

## Discussion

The COVID-19 pandemic was a worldwide challenge for all societies and everybody’s personal life. Measures of social distancing, individual safety precautions and hygiene were proven to be effective means in prevention of viral spread in most countries worldwide [14]. Also, in Germany, depending on the incidence, strict lockdowns and social distancing were imposed in order to slow down the spread of the COVID-19 infection by home quarantines, residence regulations, nationwide and regional curfews, cancellation of community events and the restriction of local and long-distance public transport. Elective health treatments have been temporarily suspended or postponed in favor of caring for affected patients and conserving human resources [15, 16].

During the COVID-19 pandemic maxillofacial trauma procedures in Germany declined by 10.7%. This applies equally to the years 2020 and 2021, between which the number of interventions fell only slightly by 2.8% which might be a consequence of the pandemic wave and their associated measures that only began in the course of 2020 and the longer lasting second lockdown in the first 4 months of 2021. The median annual number of inpatient facial injury associated diagnoses, including patients with conservative and soft tissue injury inpatient treatment, decreased by 19.7% during the pandemic. As a shift in conservative and soft tissue injury treatment from the inpatient to the outpatient sector is likely within the situation of scarce medical resources in the pandemic and the number of facial injury associated diagnoses is not necessarily related to the severity of the injury or the number patients affected, this number has to be interpreted with caution when interpreted as a surrogate for the number of underlying traumatic events.

Our results were in accordance with other studies describing a decrease of maxillofacial fractures after the COVID-19 outbreak. Salzano et al. describe a decrease in maxillofacial fractures in an Italian multicenter study [17]. De Boutray et al. show a decreased incidence of maxillofacial trauma requiring surgery in a French multicenter study [18]. This is in accordance with the experience of multiple single center studies in different European countries [11, 19–22]. Also in the USA and Australia a decrease in facial fractures was reported in single center evaluations [23–27], despite there also being studies in the USA suggesting no significant change in facial trauma incidence, maybe due to a less restrictive policy of social distancing after initial lockdown compared to European countries [28, 29]. In Asian and Middle Eastern countries, specifically in India, South Korea and Jordan a decline in maxillofacial trauma surgeries was also described [30–33]. Non-maxillofacial fractures like ankle, hip or distal radius fractures were described to show a reduced incidence as well [34–36]. Reduced trauma associated admissions to primary care centers is a worldwide known phenomenon [37, 38]. Regarding the different anatomical regions, the largest decreases were seen for lateral midfacial fractures, orbital fractures and frontal sinus fractures. In contrast, the decline of mandibular fractures was more moderate. This could be a consequence of the different etiological factors influenced by the COVID-19 pandemic, especially concerning interpersonal violence and traffic accidents, which are also influenced by geographic location, population density, economic status, and cultural differences [3]. The regional analysis of the data shows that the decline in facial fractures is a nationwide effect that is not restricted to just a few cities, regions or centers. The lack of correlation to the cumulative regional COVID-19 incidence shows that the reduction in facial fractures is not due to the regional infection load but rather to the measures taken and their social effects.

Interpersonal violence was found to be the main cause of fractures in both the mandibular and midface regions especially among young men [3, 39]. In our data the decline of fractures in young male adolescents and adults between 15 and 34 years is representing more than 50% of the overall decline of maxillofacial fracture procedures. This may indicate that during the COVID-19 pandemic 2020–2021 the reduction of interpersonal violence is one of the main reasons for the reduced incidence of maxillofacial fractures. This thesis is also supported by the rather moderate, non-significant decline of fracture reductions in women for whom physical violence is not the most prominent cause of facial fractures. This also explains a significant shift to a more balanced gender ratio [3, 39]. Several single- and multicenter studies from different countries like Australia, Finland, India and the UK show a reduction in interpersonal violence associated facial fractures and injuries while the pandemic [25, 27, 40–42]. Although there are some studies describing a higher proportion of interpersonal violence in the incidence of facial injuries in France, Italy and the USA [18, 22, 23].

As road traffic accidents are the second most common cause of facial fractures the decline in road traffic accidents involving personal injury to a historic minimum is a second mechanism explaining a decline in facial fractures in Germany [43]. This is supported by data from France, Italy, Jordan and India [18, 19, 22, 33, 40, 42].

The restriction of social and societal life naturally also has an impact on private and professional contacts and could explain a decrease in facial fractures due to sports injuries and work accidents as other important causes of fractures [39, 44]. This was also described in studies from Australia, France and South Korea [18, 25, 32].

In elder people, falls are the most common cause of facial fractures, which are insignificant in other age groups [39]. The significant increase in facial fractures in the age group of 80 years and more (+ 23.5%, *p* < 0.05) and the increase in the average age suggests a significant increase in falls, possibly as a result of social distancing, social isolation and lack of social support of older people in the context of the COVID-19 outbreak. This is consistent with results describing the negative effect of social distancing on the physical and mental health of older people during the pandemic [45]. A pandemic-associated increase in facial injuries caused by falls is described in several publications from Australia, UK, India and Italy [17, 19, 20, 25, 27, 40, 42].

This study is subject to certain limitations, which are mainly related to the dataset that was available for analysis. First, the specific reimbursed OPS codes only represent fractures treated by open reduction and internal fixation in the analyzed years. Fractures treated non-surgically are not included in the dataset. Second, the study only includes trauma cases performed during a hospital admission that led to the billing of a DRG. Patients treated as outpatients or who use other reimbursement schemes could not be included. As reimbursement of trauma cases for hospitals in the DRG system covers more than 90% of the German population, we believe, however, that the dataset covers the vast majority of treatments. Another limitation is the completeness of the dataset itself, which provides information at only an aggregated level and in limited detail without information about a specific cause or severity of the injury; therefore, a more advanced analysis including anamnestic and diagnostic information was not possible in this scale using reimbursement data. This would, however, be interesting to evaluate further the impact of the COVID-19 pandemic on maxillofacial trauma care in detail.

To the best of our knowledge, this study is the first to examine the influence of the COVID-19 pandemic on the incidence and distribution of facial fractures at a national level in Germany. It can contribute to an analysis of the influence of legislative and personal measures to combat the pandemic on the associated shift in the genesis of facial fractures. The description of positive and negative effects can contribute to the future addressing of risk groups both during and outside of pandemic situations. For this purpose, the investigation of the direct influence of the measures on the causes of facial fractures should be expanded.

## Conclusion

Trauma surgery experienced a dramatic change worldwide during the COVID-19 pandemic. This has also been observed in Germany with a drastic decline in the surgical treatment of facial fractures accompanied by a significant increase in the frequency of fractures in the COVID-19 risk group of elderly people (+ 80). Measures of social distancing and personal risk avoidance had a societal positive effect on the frequency of facial injuries. However, this is contrasted by the drastic increase in fractures in the group that is most dependent on social support and basically should rather be protected by the measures taken.

## References

[1] Wiersinga WJ, Rhodes A, Cheng AC, Peacock SJ, Prescott HC (2020) Pathophysiology, transmission, diagnosis, and treatment of Coronavirus Disease 2019 (COVID-19). JAMA 324. 10.1001/jama.2020.12839 10.1001/jama.2020.12839 32648899

[2] Onyeaka H, Anumudu CK, Al-Sharify ZT, Egele-Godswill E, Mbaegbu P (2021) COVID-19 pandemic: a review of the global lockdown and its far-reaching effects. Sci Prog 104:368504211019854. 10.1177/00368504211019854 34061685 10.1177/00368504211019854 PMC10454957

[3] Schneider D, Kammerer PW, Schon G, Dinu C, Radloff S, Bschorer R (2015) Etiology and injury patterns of maxillofacial fractures from the years 2010 to 2013 in Mecklenburg-Western Pomerania, Germany: a retrospective study of 409 patients. J Craniomaxillofac Surg 43:1948–1951. 10.1016/j.jcms.2015.06.028 26427620 10.1016/j.jcms.2015.06.028

[4] Bicsák Á, Abel D, Berbuesse A, Hassfeld S, Bonitz L (2021) Evaluation of Mandibular Fractures in a German Nationwide Trauma Center between 2015 and 2017. J Oral Maxillofac Surg 21:904–910. 10.1007/s12663-021-01513-4 10.1007/s12663-021-01513-4 PMC9474798 36274900

[5] Bonitz L, Wruck V, Peretti E, Abel D, Hassfeld S, Bicsák Á (2021) Long-term evaluation of treatment protocols for isolated midfacial fractures in a German nation-wide craniomaxillofacial trauma center 2007–2017. *Scientific Reports* 11. 10.1038/s41598-021-97858-4 10.1038/s41598-021-97858-4 PMC8440643 34521960

[6] Ellis E, el-Attar A, Moos K (1985) An analysis of 2,067 cases of zygomatico-orbital fracture. J Oral Maxillofac Surg 43:417–428. 10.1016/s0278-2391(85)80049-5 3858478 10.1016/s0278-2391(85)80049-5

[7] Iida S, Kogo M, Sugiura T, Mima T, Matsuya T (2001) Retrospective analysis of 1502 patients with facial fractures. Int J Oral Maxillofac Surg 30:286–290. 10.1054/ijom.2001.0056 11518349 10.1054/ijom.2001.0056

[8] Lentge F, Tavassol F (2024) Primary treatment of midface fractures. Die MKG-Chirurgie 17:10–17. 10.1007/s12285-023-00458-6 10.1007/s12285-023-00458-6

[9] Schneider M, Kolk A, Neff A (2023) Traumatology of the mandible. Die MKG-Chirurgie 16:193–195. 10.1007/s12285-023-00437-x 10.1007/s12285-023-00437-x

[10] Tavassol F (2024) Traumatology of the midface and the skull base. Die MKG-Chirurgie 17:1–3. 10.1007/s12285-023-00462-w 10.1007/s12285-023-00462-w

[11] Bartella AK, Halama D, Kamal M, Hahnel S, Sander AK, Pausch NC, Lethaus B (2021) Impact of COVID-19 on oral and maxillofacial surgery: preliminary results after the curfew. J Craniofac Surg 32:e305–e308. 10.1097/SCS.0000000000007062 32941222 10.1097/SCS.0000000000007062

[12] Canzi G, De Ponti E, Corradi F, Bini R, Novelli G, Bozzetti A, Sozzi D (2020) Epidemiology of Maxillo-Facial Trauma during COVID-19 Lockdown: reports from the Hub Trauma Center in Milan. Craniomaxillofacial Trauma Reconstruction 14:277–283. 10.1177/1943387520983119 34707787 10.1177/1943387520983119 PMC8543598

[13] Robert-Koch-Institut (2023) SARS-CoV-2 Infektionen in Deutschland (2023-08-24) [Data set]. *Zenodo*. 10.5281/zenodo.10836699

[14] Talic S, Shah S, Wild H, Gasevic D, Maharaj A, Ademi Z et al (2021) Effectiveness of public health measures in reducing the incidence of covid-19, SARS-CoV-2 transmission, and covid-19 mortality: systematic review and meta-analysis. BMJ. 10.1136/bmj-2021-068302 34789505 10.1136/bmj-2021-068302 PMC9423125

[15] Spiliopoulos L (2022) On the effectiveness of COVID-19 restrictions and lockdowns: Pan Metron ariston. BMC Public Health 22. 10.1186/s12889-022-14177-7 10.1186/s12889-022-14177-7 PMC9526209 36183075

[16] Bozorgmehr K, Rohleder S, Duwendag S, Mohsenpour A, Saint V, Gold AW et al (2021) Covid-19 pandemic policy monitor (COV-PPM) - European level tracking data of non-pharmaceutical interventions. Data Brief 39. 10.1016/j.dib.2021.107579 10.1016/j.dib.2021.107579 PMC8592638 34805466

[17] Salzano G, Dell’Aversana Orabona G, Audino G, Vaira LA, Trevisiol L, D’Agostino A et al (2021) Have there been any changes in the epidemiology and etiology of Maxillofacial Trauma during the COVID-19 pandemic? An Italian Multicenter Study. J Craniofac Surg 32:1445–1447. 10.1097/SCS.0000000000007253 33229987 10.1097/SCS.0000000000007253 PMC8162046

[18] de Boutray M, Kun-Darbois JD, Sigaux N, Lutz JC, Veyssiere A, Sesque A et al (2021) Impact of the COVID-19 lockdown on the epidemiology of maxillofacial trauma activity: a French multicentre comparative study. Int J Oral Maxillofac Surg 50:750–755. 10.1016/j.ijom.2020.10.005 33172710 10.1016/j.ijom.2020.10.005 PMC7648505

[19] Spallaccia F, Vellone V, Colangeli W, De Tomaso S (2022) Maxillofacial fractures in the Province of Terni (Umbria, Italy) in the last 11 years: impact of COVID-19 pandemic. J Craniofac Surg 33:e853–e858. 10.1097/SCS.0000000000008786 35882250 10.1097/SCS.0000000000008786 PMC9612422

[20] Mumtaz S, Cymerman J, Komath D (2022) Cycling-related injuries during COVID-19 lockdown: a North London experience. Craniomaxillofac Trauma Reconstr 15:46–50. 10.1177/19433875211007008 35265277 10.1177/19433875211007008 PMC8899346

[21] Kasem A, Redenski I, Oren D, Zoabi A, Srouji S, Kablan F (2022) Decline in Maxillofacial injuries during the pandemic: the Hidden Face of COVID-19. J Clin Med 12. 10.3390/jcm12010128 10.3390/jcm12010128 PMC9821158 36614929

[22] Giovannetti F, Lupi E, Di Giorgio D, Scarsella S, Oliva A, Di Fabio D et al (2022) Impact of COVID19 on Maxillofacial fractures in the Province of L’Aquila, Abruzzo, Italy. Review of 296 patients treated with statistical comparison of the two-year Pre-COVID19 and COVID19. J Craniofac Surg 33:1182–1184. 10.1097/SCS.0000000000008468 36041111 10.1097/SCS.0000000000008468 PMC9232240

[23] Stanisce L, Fisher AH, Choi BY, Newman A, Wang JL, Koshkareva Y (2022) How did the COVID-19 pandemic affect trends in Facial Trauma? Craniomaxillofac Trauma Reconstr 15:132–138. 10.1177/19433875211022574 35633770 10.1177/19433875211022574 PMC8941287

[24] Press SG (2021) What is the impact of the 2020 Coronavirus Lockdown on Maxillofacial Trauma? J Oral Maxillofac Surg 79:1329–e1321. 10.1016/j.joms.2021.01.010 10.1016/j.joms.2021.01.010 PMC7825971 33609446

[25] Nhongo SS, Sklavos A, Lee K, Chan STF, Austin S (2023) The changing face of maxillofacial trauma during the 2020 COVID-19 lockdowns in Melbourne, Australia. Oral Maxillofac Surg 27:125–130. 10.1007/s10006-022-01041-6 35066752 10.1007/s10006-022-01041-6 PMC8783956

[26] Ludwig DC, Nelson JL, Burke AB, Lang MS, Dillon JK (2021) What is the Effect of COVID-19-Related Social distancing on oral and maxillofacial trauma? J Oral Maxillofac Surg 79:1091–1097. 10.1016/j.joms.2020.12.006 33421417 10.1016/j.joms.2020.12.006 PMC7735032

[27] Hoffman GR, Walton GM, Narelda P, Qiu MM, Alajami A (2021) COVID-19 social-distancing measures altered the epidemiology of facial injury: a United Kingdom-Australia comparative study. Br J Oral Maxillofac Surg 59:454–459. 10.1016/j.bjoms.2020.09.006 33752920 10.1016/j.bjoms.2020.09.006 PMC7485452

[28] Longino ES, Landeen KC, Wessinger BC, Kimura KS, Davis SJ, Shastri KS et al (2022) Trends in Maxillofacial Trauma during COVID-19 at a level 1 Trauma Center. Ear Nose Throat J 1455613221088697. 10.1177/01455613221088697 10.1177/01455613221088697 PMC9008470 35414268

[29] Gangwani P, Harris H, Christie M, Mecca K, Barmak B, Kolokythas A (2021) Aetiology and Epidemiology of Maxillofacial Injuries during the Stay-At-Home period due to the COVID-19 pandemic: a single Center Study. J Oral Maxillofac Res 12:e4. 10.5037/jomr.2021.12404 35222871 10.5037/jomr.2021.12404 PMC8807146

[30] Kadanthode M, Chaudhary Z, Sharma P, Mohanty S, Sharma C (2023) Single Institute Audit of Maxillofacial Trauma Cases before and during COVID-19 pandemic. Craniomaxillofac Trauma Reconstr 16:102–111. 10.1177/19433875211064514 37222976 10.1177/19433875211064514 PMC10201186

[31] Bataineh AB (2023) Incidence and features of maxillofacial fractures at Jordanian tertiary hospital before, during and after the COVID-19 period. Med Oral Patol Oral Cir Bucal 28:e412–e417. 10.4317/medoral.25835 36641737 10.4317/medoral.25835 PMC10499347

[32] Lee DW, Choi SY, Kim JW, Kwon TG, Lee ST (2021) The impact of COVID-19 on the injury pattern for maxillofacial fracture in Daegu city, South Korea. Maxillofac Plast Reconstr Surg 43:35. 10.1186/s40902-021-00322-6 34515891 10.1186/s40902-021-00322-6 PMC8436019

[33] Vishal PO, Rohit, Prajapati VK, Shahi AK, Khaitan T (2022) Incidence of Maxillofacial Trauma amid COVID-19: a comparative study. J Maxillofac Oral Surg 21:420–425. 10.1007/s12663-020-01484-y 33250599 10.1007/s12663-020-01484-y PMC7680068

[34] Rydberg EM, Moller M, Ekelund J, Wolf O, Wennergren D (2021) Does the Covid-19 pandemic affect ankle fracture incidence? Moderate decrease in Sweden. Acta Orthop 92:381–384. 10.1080/17453674.2021.1907517 33821759 10.1080/17453674.2021.1907517 PMC8381968

[35] Brent L, Ferris H, Sorensen J, Valentelyte G, Kelly F, Hurson C, Ahern E (2022) Impact of COVID-19 on hip fracture care in Ireland: findings from the Irish hip fracture database. Eur Geriatr Med 13:425–431. 10.1007/s41999-021-00600-6 35064562 10.1007/s41999-021-00600-6 PMC8782703

[36] Olech J, Ciszewski M, Morasiewicz P (2021) Epidemiology of distal radius fractures in children and adults during the COVID-19 pandemic - a two-center study. BMC Musculoskelet Disord 22:306. 10.1186/s12891-021-04128-5 33771142 10.1186/s12891-021-04128-5 PMC7995382

[37] Kamine TH, Rembisz A, Barron RJ, Baldwin C, Kromer M (2020) Decrease in Trauma admissions with COVID-19 pandemic. West J Emerg Med 21:819–822. 10.5811/westjem.2020.5.47780 32726250 10.5811/westjem.2020.5.47780 PMC7390569

[38] Savla P, Wiginton Jt, Taka TM, Patchana T, Farahmandian R, Farr S et al (2021) Using the decrease in Trauma admissions during the COVID-19 pandemic to evaluate Compliance with Stay-at-home and Social Distancing guidelines. Cureus 13:e14444. 10.7759/cureus.14444 33996308 10.7759/cureus.14444 PMC8114965

[39] Juncar M, Tent PA, Juncar RI, Harangus A, Mircea R (2021) An epidemiological analysis of maxillofacial fractures: a 10-year cross-sectional cohort retrospective study of 1007 patients. BMC Oral Health 21:128. 10.1186/s12903-021-01503-5 33731083 10.1186/s12903-021-01503-5 PMC7968332

[40] Puglia FA, Hills A, Dawoud B, Magennis P, Chiu GA, Collaborators (2021) Management of oral and maxillofacial trauma during the first wave of the COVID-19 pandemic in the United Kingdom. Br J Oral Maxillofac Surg 59:867–874. 10.1016/j.bjoms.2020.12.021 34325945 10.1016/j.bjoms.2020.12.021 PMC7836223

[41] Haapanen A, Furuholm J, Uittamo J, Snall J (2022) Effect of social distancing during the COVID-19 pandemic on the occurrence of maxillofacial fractures in a Finnish Tertiary Trauma Centre. Acta Odontol Scand 80:157–160. 10.1080/00016357.2021.1979643 34597251 10.1080/00016357.2021.1979643

[42] Shenoi R, Rajguru J, Sangani S, Kolte V, Bhave I, Karmarkar J et al (2022) Changing patterns of oral & maxillofacial injuries before and during COVID-19 pandemic: a retrospective study. J Oral Biol Craniofac Res 12:651–655. 10.1016/j.jobcr.2022.07.012 35966969 10.1016/j.jobcr.2022.07.012 PMC9359753

[43] Destatis (2021) Traffic accident fatalities down 10.6% in 2020. Press Release Statistisches Bundesamt. https://www.destatis.de/EN/Press/2021/02/PE21_084_46.html. Accessed 20 August 2023

[44] Khoja YT, AlSwaji GF, Almazied MK, Alharbi AA, AlMohaini RA, Alamir MA (2021) Prevalence of sports injuries before and during COVID-19 Quarantine among adults of Riyadh, Saudi Arabia. J Pharm Res Int 165–175. 10.9734/jpri/2021/v33i35A31886

[45] Sepulveda-Loyola W, Rodriguez-Sanchez I, Perez-Rodriguez P, Ganz F, Torralba R, Oliveira DV, Rodriguez-Manas L (2020) Impact of social isolation due to COVID-19 on Health in Older people: Mental and Physical effects and recommendations. J Nutr Health Aging 24:938–947. 10.1007/s12603-020-1469-2 33155618 10.1007/s12603-020-1469-2 PMC7597423

